# Supplementary material for: Phase II single arm open label multicentre clinical trial to evaluate the efficacy and side effects of a combination of gefitinib and methotrexate to treat tubal ectopic pregnancies (GEM II): study protocol
Source: BMJ Open. 2013 Jul 18;3(7):e002902. doi: 10.1136/bmjopen-2013-002902 (PMC3717468; doi:10.1136/bmjopen-2013-002902)
Supplement: Author's manuscript [file bmjopen-2013-002902.draft_revisions.pdf]

Phase II single arm open label multicentre clinical trial to evaluate the efficacy and side effects of combination gefitinib and methotrexate to treat ectopic pregnancies (GEM II): study protocol

Journal:	*BMJ Open*
Manuscript ID:	bmjopen-2013-002902
Article Type:	Protocol
Date Submitted by the Author:	18-Mar-2013
Complete List of Authors:	Horne, Andrew<br>Skubisz, Monika<br>Doust, Ann<br>Duncan, W<br>Wallace, Euan<br>Critchley, Hilary; University of Edinburgh, Obstetrics and Gynaecology<br>Johns, Terry<br>Norman, Jane<br>Bhattacharya, Siladitya; Aberdeen University, Obstetrics and Gynaecology<br>Mollison, Jill<br>Rasmussen, Michael<br>Tong, Stephen
**Primary Subject Heading**:	Obstetrics and gynaecology
Secondary Subject Heading:	Reproductive medicine, Evidence based practice
Keywords:	REPRODUCTIVE MEDICINE, GYNAECOLOGY, General endocrinology < DIABETES & ENDOCRINOLOGY

Phase II single arm open label multicentre clinical trial to evaluate the efficacy and side effects of combination gefitinib and methotrexate to treat ectopic pregnancies (GEM II): study protocol

Andrew W Horne[1], Monika M Skubisz[2], Ann Doust[1], W Colin Duncan[1], Euan Wallace[3], Hilary OD Critchley[1], Terrance G Johns[3], Jane E Norman[1], Siladitya Bhattacharya[4], Jill Mollison[4], Michael Rassmusen[5], Stephen Tong[2]

Author affiliations

[1]MRC Centre for Reproductive Health, University of Edinburgh, Edinburgh, United Kingdom

[2]Translational Obstetrics Group, University of Melbourne, Mercy Hospital for Women, Melbourne, Australia

[3]Monash Institute of Medical Research, Clayton, Australia

[4]Obstetrics and Gynaecology, Division of Applied Clinical Sciences, University of Aberdeen, Aberdeen Maternity Hospital, Aberdeen, United Kingdom

[5]Mercy Hospital for Women, Melbourne, Australia

Correspondence to

Dr Andrew Horne, MRC Centre for Reproductive Health, Queen's Medical Research Institute, 47 Little France Crescent, Edinburgh EH16 4TJ, UK

Tel: +44 131 242 6609 Email: andrew.horne@ed.ac.uk

Key words

Ectopic pregnancy, methotrexate, gefitinib

For peer review only

Abstract

Introduction: Ectopic pregnancy (EP) is the most common life-threatening condition in gynaecology. Small EPs (pre-treatment serum human chorionic gonadotrophin [hCG] levels <1500IU/L) respond well to outpatient medical treatment with intramuscular methotrexate (MTX). Larger EPs take significant time to resolve with MTX and require multiple outpatient monitoring visits. Gefitinib is an orally-active epidermal growth factor receptor (EGFR) antagonist. In preclinical studies, we found that EP implantation sites express high levels of EGFR and that gefitinib augments MTX-induced regression of pregnancy-like tissue. We performed a Phase I toxicity study administering oral gefitinib and intramuscular MTX to 12 women with small stable EPs. The combination therapy did not cause significant toxicities and was well-tolerated. We noted that combination therapy resolved the EPs faster than MTX alone. We now describe the protocol of a larger single arm trial to estimate the efficacy and side effects of combination gefitinib and MTX to treat all stable ectopic pregnancies, including those of larger size currently considered unsuitable for medical therapy.

Methods and analysis: We propose undertaking a single-arm multicentre open label trial (in Edinburgh and Melbourne) and recruit 40 women with large stable ectopic pregnancies (pretreatment serum hCG >1500 IU/L). We will give single dose of intramuscular MTX (50 mg/m2) and oral gefitinib (250mg) daily for seven days. Our primary outcome is resolution of ectopic pregnancy within 25 days to non-pregnant hCG levels <5 IU/L. Our secondary outcome is safety and tolerability as determined by clinical/biochemical assessment. Outcomes will be compared to historical controls given methotrexate only.

Ethics and dissemination: Ethical approval has been obtained from Scotland A Research Ethics Committee (MREC 11/AL/0350), Southern Health Human Research Ethics Committee B (HREC 11180B) and the Mercy Health Human Research Ethics Committee (R12/25). Data will be presented at international conferences and published in peer-reviewed journals.

Trial registration number: ACTRN12611001056987.

For peer review only

Article summary

Article focus

Protocol of a study to determine:

- Is combination therapy with methotrexate and gefitinib more effective at resolving ectopic pregnancies than methotrexate alone?
- Is combination therapy with methotrexate and gefitinib safe and well-tolerated?

Key messages

- Small stable ectopic pregnancies respond well to treatment with intramuscular methotrexate.
- Larger ectopic pregnancies require multiple hospital visits to resolve with methotrexate and often require surgery.
- Novel combination therapy of methotrexate and the oral EGFR antagonist, gefitinib, could reduce the number of hospital visits required to resolve larger ectopic pregnancies.

For peer review only

Strengths and limitations of this study

- This is a Phase II exploratory efficacy trial, and will be the "first in man" to examine the efficacy of gefitinib and methotrexate to treat larger ectopic pregnancies.
- This is a 'single arm' trial comparing outcome with historical controls. The data will be used to inform a future large multicentre randomised controlled trial comparing combination therapy to surgery for treatment of ectopic pregnancies.
- The combination therapy described also has potential use in other pregnancy disorders where medical regression of placental tissue could be useful e.g. molar disease, retained products of conception after miscarriage, and regression of placenta accrete post-partum.

For peer review only

Introduction

Ectopic pregnancy (EP) is the most common life-threatening condition in modern gynaecology in both the developed and developing world (Sivalingham et al. 2011; Wilkinson and Jurkovic, 2011). Small EPs (pre-treatment serum human chorionic gonadotrophin [hCG] levels <1500IU/L) respond well to outpatient medical treatment with an intramuscular injection of methotrexate (MTX). Indeed, it has been recently suggested that these small ectopic pregnancies could be managed safely, and equally efficiently, by expectant management without medical intervention (Mavrelos et al, 2013). In contrast, for larger EPs (~60% of total EPs) emergency laparoscopic surgical excision (with its inherent risks of damage to visceral organs) remains the most effective treatment (Mol et al. 2008). Larger EPs take a significant time to resolve with MTX and require multiple outpatient monitoring visits. There therefore exists a need for more effective medical treatments for larger EPs to reduce the need for emergency surgery and reduce the time to resolution associated with MTX management.

Gefitinib is an orally active epidermal growth factor receptor (EGFR) antagonist licensed to treat non-small cell lung cancer (Herbst et al. 2004). In preclinical studies, we found that EP implantation sites express high levels of EGFR and that gefitinib augments MTX-induced regression of pregnancy-like tissue (Nilsson et al. 2010). To translate this into clinical care, we performed a Phase I single-arm open-label dose-escalation study administering a combination of 250mg oral gefitinib (one dose [n=3], three daily doses [n=3], seven daily doses [n=6]) and

For peer review only - http://bmjopen.bmj.com/site/about/guidelines.xhtml

intramuscular MTX (50 mg/m2) to 12 women with EPs. The combination therapy did not cause any significant toxicities and was well tolerated. We noted that resolution (fall in serum hCG to <5IU/L) with combination therapy was faster than the median time for EPs to resolve with MTX alone when compared to contemporaneous controls (21 versus 32 days).

Objectives

The objective of this trial is to evaluate the efficacy and side effects of combination gefitinib and MTX to treat larger ectopic pregnancies (pretreatment hCG >1500), including those currently considered unsuitable for medical therapy.

For peer review only

Methods and analysis

Study design

Phase II single-arm multicentre open label trial (Edinburgh and two sites in Melbourne).

Subjects

40 women with large stable ectopic pregnancies.

Study settings

We will recruit patients from gynaecology departments within NHS Lothian (UK), and Southern Health and Mercy Health networks in Melbourne, Australia.

Sample size

We have calculated the sample size required to estimate the true treatment effect with 90% confidence that the lower limit is at least 90%. We have assumed for the sake of these calculations that the true treatment efficacy of methotrexate/gefitinib in treating these larger ectopic pregnancies is 95%. We have chosen in this scenario, a sample size of 36 to ensure the lower limit of the 90% CI will exceed 90% (assuming a treatment effect of 95%). Our sample size of 40 will thus ensure we have sufficient precision in estimation of the true treatment effect, allowing 10% loss to follow up. We believe that this is a reasonable basis on which to conduct this initial efficacy study, of methotrexate/gefitinib in the treatment of larger ectopic pregnancies that are currently treated with surgery.

For peer review only

Inclusion criteria

Women aged aged between 18-45; serum hCG of >1500 IU/L; ultrasound diagnosis of an abnormal mass outside the uterine cavity; no clinical evidence of intra-abdominal bleeding; no pallor; no guarding/rigidity on abdominal examination; stable blood pressure and heart rate; normal haemoglobin on full blood examination at day 1.

Exclusion criteria

Women with a history of any significant pulmonary disease; abnormal liver/renal/haematological indices; significant pre-existing dermatological conditions; significant pre-existing gastrointestinal medical illnesses; Japanese ethnicity.

Participant enrolment

All gynaecology consultants within NHS Lothian (UK), Southern Health and Mercy Health (both Australia) will be sent a letter informing them of the study and requesting permission to approach their patients. The clinical research team in NHS Lothian, Southern Health and Mercy Health will approach eligible women, provide them with patient information sheets and offer them the opportunity to discuss the trial, and obtain informed consent. Consent will only be taken once the patient has had ample time to read the patient information sheet and had her questions answered.

For peer review only

Intervention

Eligible women will be given a single dose intramuscular methotrexate (50 mg/m2) injection with seven daily doses oral gefitinib (250 mg). The gefitinib will be started on the same date as the methotrexate injection is given.

Data collection

Data storage

A log with the patients' name and date of birth will be kept along with their unique study number in a separate file. All the data generated from the study will be stored in an anonymised form in a bespoke database, which will also be password protected. Only anonymised information will be stored on this, and participants will only be identifiable by their study number. All paperwork will be kept in a locked filing cabinet in a locked office. All data will be stored on university server (University of Edinburgh) on a password-protected computer with limited access to the research team, in accordance with the Data Protection Act (UK).

Screening

A member of the research team will carry out a screening visit to assess eligibility. All data will be recorded on a case record form and transferred to a secure database.

Participant log

For peer review only

The clinical research team will keep an electronic log of women who fulfil the eligibility criteria, women who are invited to participate in the study, women recruited and women who leave the trial early. Reasons for non-recruitment (eg, non-eligibility, refusal to participate, administrative error) will also be recorded. We will attempt to collect reasons for non-participation from women who decline to take part after previously providing contact details. During the course of the study, we will document reasons for withdrawal from the study and loss to follow-up.

Assessments

To monitor treatment response, we will follow protocols used clinically for single dose methotrexate. Serum hCG levels will be measured on days 4, 7 and 11, then weekly until hCG levels drop to non-pregnant levels (<5 IU/L). Participants will be contacted at 3 and 6 months post treatment to document return of menstrual cycles and any subsequent pregnancies. To monitor safety and tolerability, women will be assessed clinically (history) and biochemically (haematological, renal and liver function tests) on days 4, 7 and 11, then weekly until the ectopic pregnancy has resolved (see below).

Primary outcome

Our primary outcome is resolution of ectopic pregnancy within 25 days. Resolution is defined by serum hCG levels (the current clinical marker to monitor treatment response) falling to non-pregnant levels (<5 IU/L). We have selected our primary outcome based on the data from our Phase I trial that suggested that duration of resolution with combination therapy was faster than

For peer review only

the median time for EPs to resolve with MTX alone (21 versus 32 days). For practical purposes, we have chosen a primary outcome of resolution within 25 days rather than 21 days because patients are not normally seen between 18 and 25 days post treatment (our trial participants are followed up weekly from day 11 to conform to standard monitoring protocols for treatment with MTX) (RCOG 2004).

Secondary outcome

Safety and tolerability as determined by clinical and biochemical assessment. Both methotrexate and gefitinib have the potential to affect haematological, renal and liver function.

Proposed analyses

Given this is a single arm efficacy trial, the majority of the data will be expressed as descriptive statistics.

Ethics and dissemination

Ethical approval has been obtained from the Scotland A Research Ethics Committee (LREC 12/SS/0005) (UK), the Southern Health Human Research Ethics Committee B (SH HREC 11180B) and the Mercy Health Human Research Ethics Committee (R12/25) (both Australia). Data will be presented at international conferences and published in peer-reviewed journals. We will make the information obtained from the study available to the public through national bodies and charities (e.g. Ectopic Pregnancy Trust).

For peer review only

Adverse events

Participants will collect information about adverse events in their treatment diaries. However, they will be instructed to contact the clinical research team at any time after consenting to join the trial if they have an event that requires hospitalisation or an event that results in persistent or significant disability or incapacity. Any serious adverse events that occur after joining the trial will be reported in detail in the participant's medical notes, followed up until resolution of the event and reported to the ACCORD Research Governance (http://www.accord.ed.ac.uk) and QA Office based at the University of Edinburgh, or the Southern Health/Mercy Health Human Research Ethics Committees and Therapeutic Goods Administration of Australia's Office of Scientific Evaluation immediately or within 24-72 hours.

For peer review only

Discussion

If effective, we believe that this combination (gefitinib and methotrexate) could become standard of care for stable ectopic pregnancies. The combination also has potential use in other pregnancy disorders. There may be other important conditions where medical regression of pregnancy tissue could be useful, for example, women with complete molar pregnancies and persistent molar disease, women with retained products of conception after miscarriage, women with placenta accrete postpartum (to avoid hysterectomy), and therapeutic termination of pregnancy.

However, we do not anticipate that this will be the final trial to determine whether further exploration of this area is worthwhile. We hope that the study will generate sufficient "signal" that gefitinib and methotrexate may be effective, to support a funding application for a larger trial with a comparative group. Such a trial could be designed as an "equivalence" trial in terms of treatment efficacy between surgery and the gefitinib/methotrexate comparison. It would aim to test the hypothesis that gefitinib/methotrexate was superior in a range of outcomes prioritised by consumer groups and clinicians. We anticipate that these outcomes could include: time in hospital, time to resumption of normal activities, SF-36 at intervals after treatment, and patient satisfaction scores. Outcomes of a subsequent pregnancy are also important but would require long-term follow up studies. We anticipate that focus groups and surveys of patients and clinicians would be required to define the outcomes (other than efficacy) of these studies.

For peer review only

References

Herbst RS, Fukuoka M, Baselga J. Gefitinib-a novel targeted approach to treating cancer. Nat Rev Cancer 2004;4:956-65.

Jurkovic D, Wilkinson H. Diagnosis and management of ectopic pregnancy. BMJ 2011 342:d3397.

Mavrelos D, Nicks H, Jamil A, Hoo W, Jauniaux E, Jurkovic D. Efficacy and safety of a clinical protocol for expectant management of selected women diagnosed with a tubal ectopic pregnancy. Ultrasound Obstet Gynecol 2013 [epub ahead of print].

Mol F, Mol BW, Ankum WM, van der Veen F, Hajenius PJ. Current evidence on surgery, systemic methotrexate and expectant management in the treatment of tubal ectopic pregnancy: a systematic review and meta-analysis. Hum Reprod Update 2008;14:309-19.

Nilsson U, Gano YE, Johns TG, et al. Epidermal Growth Factor Receptor Inhibition as a Novel Tablet-Based Treatment to Medically Cure Ectopic Pregnancy. Reprod Sci 2010; 17(Supplement):157A.

RCOG. The management of tubal pregnancy. Green top Guideline No 21, published in May 2004. http://www.rcog.org.uk/files/rcog-corp/GTG21_230611.pdf

For peer review only

Sivalingam VN, Duncan WC, Kirk E, Shephard LA, Horne AW. Diagnosis and management of ectopic pregnancy. J Fam Plann Reprod Health Care. 2011 37(4):231-40.

For peer review only

Funding

This work is supported by an NHMRC Grant (#1008276) to ST, TJ and EW, and an MRC Centenary Award (G0802808) to AH. The funders will have no role in the study design; collection, management, analysis and interpretation of data; writing of the report; and the decision to submit the report for publication.

Competing interests

AH is funded by an MRC Clinician Scientist Fellowship and MRC Centenary Award (G0802808) and holds grants from the Chief Scientist's Office Scotland (CZH/4/688) (HC co-investigator) and Wellbeing of Women. HC holds an MRC DCS Grant (G003611), an MRC Centre Grant (G1002033) and research collaboration funding from Bayer Pharma AG. UN, TJ, and ST are joint holders of patents that relate to the use of EGFR inhibition in treating ectopic pregnancies. AH and HC hold the University of Edinburgh Patent 'Identification of Ectopic Pregnancies' number 0712801.0.

Authors' contributions

AH, ST: research, contribution of original material, editing and approval of final manuscript; AD, MS: contribution of original material, editing and approval of final manuscript; MS, MR, CD, EW, HC, TJ, SB, JM, JN: editing and approval of final manuscript.

For peer review only

Figure legend

Figure 1. Flow of participants through the study.

For peer review only

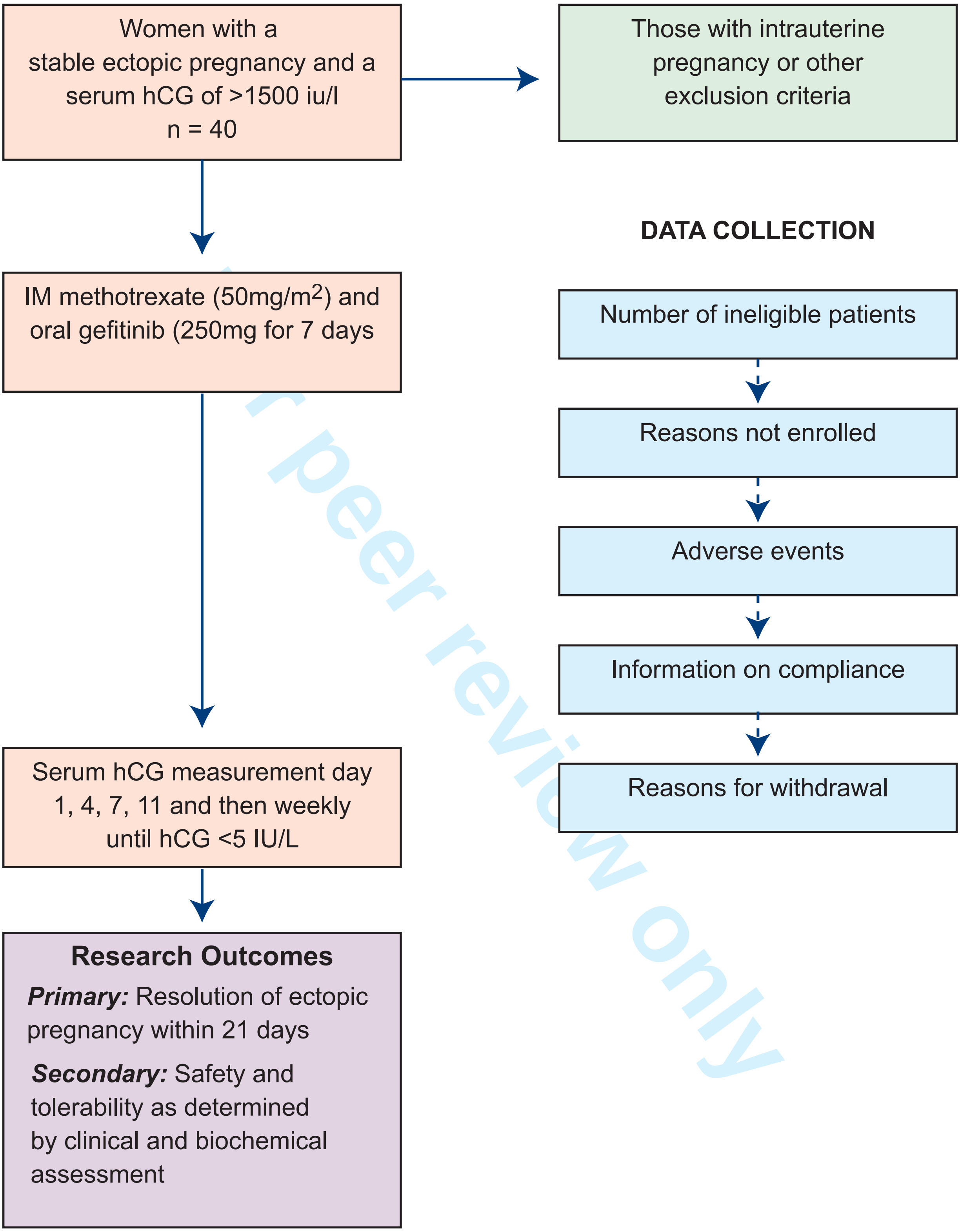

For peer review only

Phase II single arm open label multicentre clinical trial to evaluate the efficacy and side effects of combination gefitinib and methotrexate to treat ectopic pregnancies (GEM II): study protocol

Journal:	*BMJ Open*
Manuscript ID:	bmjopen-2013-002902.R1
Article Type:	Protocol
Date Submitted by the Author:	09-May-2013
Complete List of Authors:	Horne, Andrew<br>Skubisz, Monika<br>Doust, Ann<br>Duncan, W<br>Wallace, Euan<br>Critchley, Hilary; University of Edinburgh, Obstetrics and Gynaecology<br>Johns, Terry<br>Norman, Jane<br>Bhattacharya, Siladitya; Aberdeen University, Obstetrics and Gynaecology<br>Mollison, Jill<br>Rasmussen, Michael<br>Tong, Stephen
**Primary Subject Heading**:	Obstetrics and gynaecology
Secondary Subject Heading:	Reproductive medicine, Evidence based practice, Pharmacology and therapeutics
Keywords:	REPRODUCTIVE MEDICINE, GYNAECOLOGY, General endocrinology < DIABETES & ENDOCRINOLOGY

SCHOLARONE™
Manuscripts

Phase II single arm open label multi-centre clinical trial to evaluate the efficacy and side effects of combination gefitinib and methotrexate to treat tubal ectopic pregnancies (GEM II): study protocol

Andrew W Horne[1], Monika M Skubisz[2], Ann Doust[1], W Colin Duncan[1], Euan Wallace[3], Hilary OD Critchley[1], Terrance G Johns[3], Jane E Norman[1], Siladitya Bhattacharya[4], Jill Mollison[4], Michael Rassmusen[5], Stephen Tong[2]

Author affiliations

[1]MRC Centre for Reproductive Health, University of Edinburgh, Edinburgh, United Kingdom

[2]Translational Obstetrics Group, University of Melbourne, Mercy Hospital for Women, Melbourne, Australia

[3]Monash Institute of Medical Research, Clayton, Australia

[4]Obstetrics and Gynaecology, Division of Applied Clinical Sciences, University of Aberdeen, Aberdeen Maternity Hospital, Aberdeen, United Kingdom

[5]Mercy Hospital for Women, Melbourne, Australia

Correspondence to

Dr Andrew Horne, MRC Centre for Reproductive Health, Queen's Medical Research Institute, 47 Little France Crescent, Edinburgh EH16 4TJ, UK

Tel: +44 131 242 6609 Email: andrew.horne@ed.ac.uk

Key words

Ectopic pregnancy, methotrexate, gefitinib

For peer review only

Abstract

Introduction: Tubal ectopic pregnancy (tEP) is the most common life-threatening condition in gynaecology. TEPs with pre-treatment serum human chorionic gonadotrophin [hCG] levels <1000IU/L respond well to outpatient medical treatment with intramuscular methotrexate (MTX). TEPs with hCG >1000IU/L take a significant time to resolve with MTX and require multiple outpatient monitoring visits. Gefitinib is an orally active epidermal growth factor receptor (EGFR) antagonist. In preclinical studies, we found that EP implantation sites express high levels of EGFR and that gefitinib augments MTX-induced regression of pregnancy-like tissue. We performed a Phase I toxicity study administering oral gefitinib and intramuscular MTX to 12 women with tEPs. The combination therapy did not cause significant toxicities and was well tolerated. We noted that combination therapy resolved the tEPs faster than MTX alone. We now describe the protocol of a larger single arm trial to estimate the efficacy and side effects of combination gefitinib and MTX to treat stable tEPs with hCG 1,000-10,000IU/L

Methods and analysis: We propose undertaking a single-arm multicentre open label trial (in Edinburgh and Melbourne) and recruit 28 women with tEPs (pretreatment serum hCG 1,000-10,000IU/L). We will give a single dose of intramuscular MTX (50 mg/m2) and oral gefitinib (250mg) daily for seven days. Our primary outcome is resolution of ectopic pregnancy to non-pregnant hCG levels <15IU/L without requirement for surgery. Our secondary outcomes are comparison of time to resolution against historical controls given MTX only, and safety and tolerability as determined by clinical/biochemical assessment.

For peer review only

Ethics and dissemination: Ethical approval has been obtained from Scotland A Research Ethics Committee (MREC 11/AL/0350), Southern Health Human Research Ethics Committee B (HREC 11180B) and the Mercy Health Human Research Ethics Committee (R12/25). Data will be presented at international conferences and published in peer-reviewed journals.

Trial registration number: ACTRN12611001056987.

For peer review only

Article summary

Article focus

Protocol of a study to determine:

- Is combination therapy with methotrexate and gefitinib effective at resolving tEPs?
- Is combination therapy with methotrexate and gefitinib safe and well tolerated?

Key messages

- tEPs with hCG levels <1,000IU/L respond well to treatment with intramuscular methotrexate.
- tEPs with hCG levels >1,000IU/L require multiple hospital visits to resolve with methotrexate and often require surgery.
- Novel combination therapy of methotrexate and the oral EGFR antagonist, gefitinib, could reduce the number of hospital visits required to resolve tEPs with hCG levels >1,000IU/L.

For peer review only

Strengths and limitations of this study

- This is a Phase II exploratory efficacy trial, and will be the "first in man" to examine the efficacy of gefitinib and methotrexate to treat tEPs with hCG levels >1,000IU/L
- This is a 'single arm' trial. The data will be used to inform a future large multicentre randomised controlled trial comparing combination therapy to conventional management of tEPs.
- The combination therapy described also has potential use in other pregnancy disorders where medical regression of placental tissue could be useful e.g. molar disease and regression of placenta accrete post-partum.

For peer review only

Introduction

Tubal ectopic pregnancy (tEP) is the most common life-threatening condition in modern gynaecology in both the developed and developing world (Sivalingham et al. 2011; Wilkinson and Jurkovic, 2011). TEPs with pre-treatment serum human chorionic gonadotrophin [hCG] levels <1,000IU/L respond well to outpatient medical treatment with an intramuscular injection of methotrexate (MTX). Indeed, it has been suggested that these tEPs could be managed safely, and equally efficiently by expectant management without medical intervention (RCOG, 2010; Mavrelos et al, 2013; van Mello et al, 2013). In contrast, single-dose MTX is only cost–effective in women with serum hCG concentrations <1,500 IU/l (Mol et al. 2008). In tEPs with higher hCG levels (>60% of total tEPs), emergency laparoscopic surgical excision (with its inherent risks of damage to visceral organs) remains the most effective treatment. TEPs with higher hCG levels take a significant time to resolve with MTX and require multiple outpatient monitoring visits. There therefore exists a need for more effective medical treatments for tEPs with higher hCG levels to reduce the need for emergency surgery and reduce the time to resolution associated with MTX management.

Gefitinib is an orally active epidermal growth factor receptor (EGFR) antagonist licensed to treat non-small cell lung cancer (Herbst et al. 2004). In preclinical studies, we found that EP implantation sites express high levels of EGFR and that gefitinib augments MTX-induced regression of pregnancy-like tissue (Nilsson et al. 2010). To translate this into clinical care, we performed a Phase I single-arm open-label dose-escalation study administering a combination of 250mg oral

For peer review only

gefitinib (one dose [n=3], three daily doses [n=3], seven daily doses [n=6]) and intramuscular MTX (50 mg/m2) to 12 women with tEPs. The combination therapy did not cause any significant toxicities, and was well tolerated. We noted that resolution (fall in serum hCG to <15IU/L) with combination therapy was faster than the median time for tEPs to resolve with MTX alone when compared to contemporaneous controls (21 versus 32 days).

Objectives

The objective of this trial is to evaluate the efficacy and side effects of combination gefitinib and MTX to treat tEPs (hCG 1,000-10,000IU/L).

For peer review only

Methods and analysis

Study design

Phase II single-arm multi-centre open label trial (Edinburgh and two sites in Melbourne).

Subjects

28 women with tEPs with hCG levels 1,000-10,000IU/L.

Study settings

We will recruit patients from gynaecology departments within NHS Lothian (UK), and Southern Health and Mercy Health networks in Melbourne, Australia.

Sample size

We have calculated the sample size using A'Hern's formula for Phase II one-stage designs (A'Hern 2001). For treatment of tEPs with hCG levels 1,000-10,000IU/L by methotrexate/gefitinib to be considered effective, we would expect a success rate of at least 90%. However, a success rate of 70% or less would be considered unacceptable. With 80% power and a 5% level of significance, 28 patients are required to enable us to assess whether the proportion of patients with a successful outcome to treatment is ≤ 70% or ≥90%. If 24, or more, patients have a successful outcome, we can reject the hypothesis that the true efficacy of methotrexate/gefitinib is ≤ 70% and progress to a phase II trial.

.

For peer review only

Inclusion criteria

Women aged between 18-45 years; pre-treatment serum hCG of 1,000-10,000IU/L (rising or static); ultrasound diagnosis of definite tEP (extrauterine gestational sac with yolk sac and/or embryo, with or without cardiac activity) or probable tEP (inhomogeneous adnexal mass or extrauterine sac-like structure) (Barnhart et al, 2011) performed by a clinical team of trained, qualified and experienced ultrasonographers; no clinical evidence of intra-abdominal bleeding; no pallor; no guarding/rigidity on abdominal examination; stable blood pressure and heart rate; haemoglobin on full blood examination at day 1 between 100-165 g/L) .

Exclusion criteria

Women with a pregnancy of unknown location; evidence of a significant intra-abdominal bleed on ultrasound defined by free fluid above the uterine fundus or the surrounding ovary (Fauconnier et al, 2007); women with a history of any significant pulmonary disease; abnormal liver/renal/haematological indices; significant pre-existing dermatological conditions; significant pre-existing gastrointestinal medical illnesses; Japanese ethnicity.

Participant enrolment

All gynaecology consultants within NHS Lothian (UK), Southern Health and Mercy Health (both Australia) will be sent a letter informing them of the study and requesting permission to approach their patients. The clinical research team in NHS Lothian, Southern Health and Mercy Health will approach eligible women, provide them with patient information sheets and offer them the opportunity to

For peer review only

discuss the trial, and obtain informed consent. Consent will only be taken once the patient has had ample time to read the patient information sheet and had her questions answered.

Intervention

Eligible women will be given a single dose intramuscular methotrexate (50 mg/m2) injection with seven daily doses oral gefitinib (250 mg). The gefitinib will be started on the same date as the methotrexate injection is given.

Data collection

Data storage

A log with the patients' name and date of birth will be kept along with their unique study number in a separate file. All the data generated from the study will be stored in an anonymised form in a bespoke database, which will also be password protected. Only anonymised information will be stored on this, and participants will only be identifiable by their study number. All paperwork will be kept in a locked filing cabinet in a locked office. All data will be stored on university server (University of Edinburgh) on a password-protected computer with limited access to the research team, in accordance with the Data Protection Act (UK).

Screening

For peer review only

A member of the research team will carry out a screening visit to assess eligibility. All data will be recorded on a case record form and transferred to a secure database.

Participant log

The clinical research team will keep an electronic log of women who fulfil the eligibility criteria, women who are invited to participate in the study, women recruited and women who leave the trial early. Reasons for non-recruitment (eg, non-eligibility, refusal to participate, administrative error) will also be recorded. We will attempt to collect reasons for non-participation from women who decline to take part after previously providing contact details. During the course of the study, we will document reasons for withdrawal from the study and loss to follow-up.

Assessments

To monitor treatment response, we will follow protocols used clinically for medical management with methotrexate. Serum hCG levels will be measured on days 4, 7 and 11, then weekly until hCG levels drop to non-pregnant levels (<15 IU/L). Medical management will be discontinued and patients will undergo surgery based on their response to methotrexate and clinical picture (e.g. clinical evidence of intra-abdominal bleeding) following standard clinical paradigms documented by the assessing clinician. Participants will be contacted at 3 and 6 months post treatment to document return of menstrual cycles and any subsequent pregnancies. To monitor safety and tolerability, women will be assessed clinically (history) and biochemically (haematological, renal and liver

For peer review only

function tests) on days 4 and 7 (or if elevated, until return to normal physiological levels).

Primary outcome

Our primary outcome is resolution of tEP without requirement for surgery. Resolution is defined by serum hCG levels (the current clinical marker to monitor treatment response) falling to non-pregnant levels (hCG <15IU/L). We have selected our primary outcome based on the data from our Phase I trial where two patients recruited with pretreatment hCG levels >1,000IU/L required surgery and previously published data (Menon et al, 2007). We are using a cut-off of <15IU/L, which corresponds to a negative urinary pregnancy test using the most sensitive assays.

Secondary outcome

(i) Time to resolution (categorical variable) compared to historical controls of similar pre-treatment serum hCG levels (identified by an individual blinded to the study).

(ii) Safety and tolerability as determined by clinical and biochemical assessment. Both methotrexate and gefitinib have the potential to affect haematological, renal and liver function.

Proposed analyses

Given this is a single arm efficacy trial, the majority of the data will be expressed as descriptive statistics.

For peer review only

Ethics and dissemination

Ethical approval has been obtained from the Scotland A Research Ethics Committee (LREC 12/SS/0005) (UK), the Southern Health Human Research Ethics Committee B (SH HREC 11180B) and the Mercy Health Human Research Ethics Committee (R12/25) (both Australia). Data will be presented at international conferences and published in peer-reviewed journals. We will make the information obtained from the study available to the public through national bodies and charities (e.g. Ectopic Pregnancy Trust).

Adverse events

Participants will collect information about adverse events in their treatment diaries. However, they will be instructed to contact the clinical research team at any time after consenting to join the trial if they have an event that requires hospitalisation or an event that results in persistent or significant disability or incapacity. Any serious adverse events that occur after joining the trial will be reported in detail in the participant's medical notes, followed up until resolution of the event and reported to the ACCORD Research Governance (http://www.accord.ed.ac.uk) and QA Office based at the University of Edinburgh, or the Southern Health/Mercy Health Human Research Ethics Committees and Therapeutic Goods Administration of Australia's Office of Scientific Evaluation immediately or within 24-72 hours.

For peer review only

Discussion

If effective, we believe that this combination (gefitinib and methotrexate) could become standard of care for stable tEPs. The combination also has potential use in other pregnancy disorders. There may be other important conditions where medical regression of pregnancy tissue could be useful, for example, women with complete molar pregnancies and persistent molar disease, and women with placenta accrete postpartum (to avoid hysterectomy).

Regarding the safety of gefitinib, data from post marketing surveillance representing over 92,000 patients exists, and has shown that EGFR inhibitors are well tolerated and largely free of serious side effects (FDA report: (Cohen et al, 2004) . Of note, the data on tolerability is based on patients taking gefitinib daily on an ongoing, indefinite basis, after primary treatment of a cancer. Diarrhea and skin rash are the most common side effects (20-30%). The skin rash, described as acneiform, can be severe, but is generally self-limited. Skin rashes occur within a month of initiation of treatment, but rarely in the first week. Interstitial lung disease (ILD) is a very rare but serious side effect of gefitinib. It is a thickening of the lung parenchyma that can be fatal in a third of cases. Of the 31,045 patients in the USA who took gefitinib (reported to the FDA), 84 developed ILD (0.3%). We plan to administer seven 250mg gefitinib tablets, one daily for only seven days, in addition to methotrexate. This is an extremely short duration of treatment compared with gefitinib's current marketing indications and existing data usage. We would not expect this short course to have an adverse long-term effect on fertility but we will be assessing participants 3 and 6

For peer review only

months post treatment to document return of menstrual cycles and any subsequent pregnancies.

We do not anticipate that this will be the final trial to determine whether further exploration of combination therapy with gefitinib and methotrexate is worthwhile. We hope that the study will generate sufficient "signal" that gefitinib and methotrexate may be effective and safe, to support a funding application for a larger trial with a comparative group. Such a trial could be designed as an "equivalence" trial in terms of treatment efficacy between conventional management and the gefitinib/methotrexate comparison. It would aim to test the hypothesis that gefitinib/methotrexate was superior in a range of outcomes prioritised by consumer groups and clinicians. We anticipate that these outcomes could include: time to resumption of normal activities, SF-36 at intervals after treatment, and patient satisfaction scores. Outcomes of a subsequent pregnancy are also important but would require long-term follow up studies. We anticipate that focus groups and surveys of patients and clinicians would be required to define the outcomes (other than efficacy) of these studies.

References

A'Hern RP. Sample size tables for exact single stage Phase II designs. Stat Med 2001 20:859-866.

Barnhart K, van Mello NM, Bourne T, Kirk E, Van Calster B, Bottomley C, Chung K, Condous G, Goldstein S, Hajenius PJ, Mol BW, Molinaro T, O'Flynn O'Brien KL,

Husicka R, Sammel M, Timmerman D. Pregnancy of unknown location: a consensus statement of nomenclature, definitions, and outcome. Fertil Steril. 2011 95(3):857-66.

Cohen MH, Williams GA, Sridhara R, Chen G, McGuinn WD Jr, Morse D, Abraham S, Rahman A, Liang C, Lostritto R, Baird A, Pazdur R. United States Food and Drug Administration Drug Approval summary: Gefitinib (ZD1839; Iressa) tablets. Clin Cancer Res. 2004 10(4):1212-8.

Fauconnier A, Mabrouk A, Salomon LJ, Bernard JP, Ville Y. Ultrasound assessment of haemoperitoneum in ectopic pregnancy: derivation of a prediction model. World J Emerg Surg. 2007 2:23.

Herbst RS, Fukuoka M, Baselga J. Gefitinib-a novel targeted approach to treating cancer. Nat Rev Cancer 2004;4:956-65.

Jurkovic D, Wilkinson H. Diagnosis and management of ectopic pregnancy. BMJ 2011 342:d3397.

Mavrelos D, Nicks H, Jamil A, Hoo W, Jauniaux E, Jurkovic D. Efficacy and safety of a clinical protocol for expectant management of selected women diagnosed with a tubal ectopic pregnancy. Ultrasound Obstet Gynecol 2013 [epub ahead of print].

For peer review only

Menon S, Colins J, Barnhart KT. Establishing a human chorionic gonadotropin cutoff to guide methotrexate treatment of ectopic pregnancy: a systematic review. Fertil Steril. 2007 87(3):481-4.

Mol F, Mol BW, Ankum WM, van der Veen F, Hajenius PJ. Current evidence on surgery, systemic methotrexate and expectant management in the treatment of tubal ectopic pregnancy: a systematic review and meta-analysis. Hum Reprod Update 2008;14:309-19.

Nilsson U, Gano YE, Johns TG, et al. Epidermal Growth Factor Receptor Inhibition as a Novel Tablet-Based Treatment to Medically Cure Ectopic Pregnancy. Reprod Sci 2010; 17(Supplement):157A.

RCOG. The management of tubal pregnancy. Green top Guideline No 21, published in May 2004 (reviewed 2010). http://www.rcog.org.uk/files/rcog-corp/GTG21_230611.pdf

Sivalingam VN, Duncan WC, Kirk E, Shephard LA, Horne AW. Diagnosis and management of ectopic pregnancy. J Fam Plann Reprod Health Care. 2011 37(4):231-40.

van Mello NM, Mol F, Verhoeve HR, van Wely M, Adriaanse AH, Boss EA, Dijkman AB, Bayram N, Emanuel MH, Friederich J, van der Leeuw-Harmsen L, Lips JP, Van Kessel MA, Ankum WM, van der Veen F, Mol BW, Hajenius PJ. Methotrexate or

For peer review only

expectant management in women with an ectopic pregnancy or pregnancy of unknown location and low serum hCG concentrations? A randomized comparison. Hum Reprod. 2013 28(1):60-7.

For peer review only

Funding

This work is supported by an NHMRC Grant (#1008276) to ST, TJ and EW, and an MRC Centenary Award (G0802808) to AH. The funders will have no role in the study design; collection, management, analysis and interpretation of data; writing of the report; and the decision to submit the report for publication.

Competing interests

AH is funded by an MRC Clinician Scientist Fellowship and MRC Centenary Award (G0802808) and holds grants from the Chief Scientist's Office Scotland (CZH/4/688) (HC co-investigator) and Wellbeing of Women. HC holds an MRC DCS Grant (G003611), is a co-applicant of an MRC Centre Grant (G1002033) and has research collaboration funding from Bayer Pharma AG. UN, TJ, and ST are joint holders of patents that relate to the use of EGFR inhibition in treating ectopic pregnancies.

Authors' contributions

AH, ST: research, contribution of original material, editing and approval of final manuscript; AD, MS: contribution of original material, editing and approval of final manuscript; MS, MR, CD, EW, HC, TJ, SB, JM, JN: editing and approval of final manuscript.

For peer review only

Figure legend

Figure 1. Flow of participants through the study.

For peer review only

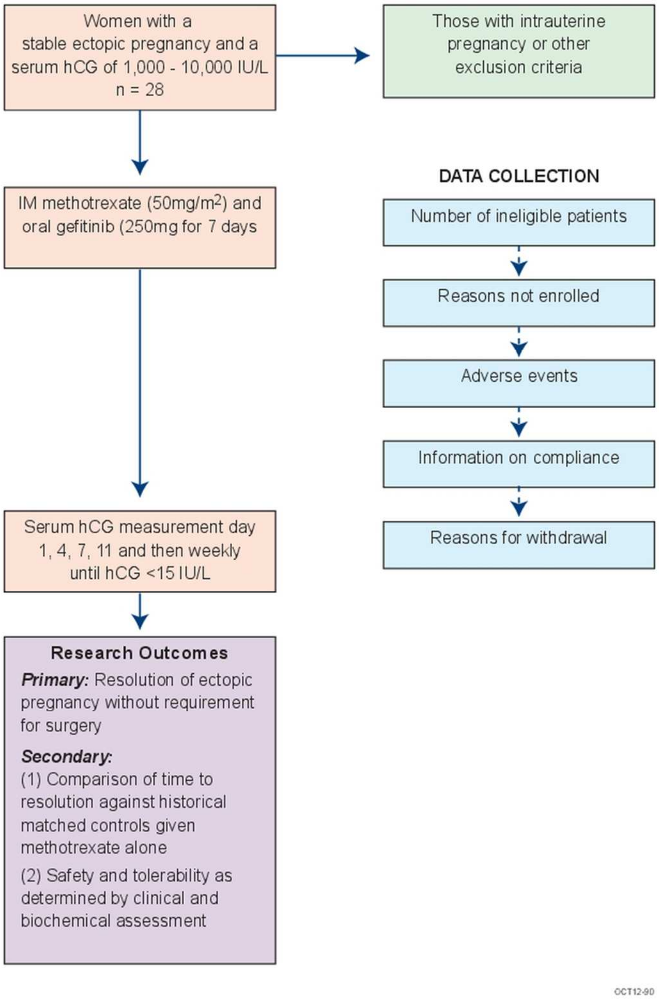

90x127mm (300 x 300 DPI)

Phase II single arm open label multi-centre clinical trial to evaluate the efficacy and side effects of combination gefitinib and methotrexate to treat tubal ectopic pregnancies (GEM II): study protocol

Andrew W Horne[1], Monika M Skubisz[2], Ann Doust[1], W Colin Duncan[1], Euan Wallace[3], Hilary OD Critchley[1], Terrance G Johns[3], Jane E Norman[1], Siladitya Bhattacharya[4], Jill Mollison[4], Michael Rassmusen[5], Stephen Tong[2]

Author affiliations

[1]MRC Centre for Reproductive Health, University of Edinburgh, Edinburgh, United Kingdom

[2]Translational Obstetrics Group, University of Melbourne, Mercy Hospital for Women, Melbourne, Australia

[3]Monash Institute of Medical Research, Clayton, Australia

[4]Obstetrics and Gynaecology, Division of Applied Clinical Sciences, University of Aberdeen, Aberdeen Maternity Hospital, Aberdeen, United Kingdom

[5]Mercy Hospital for Women, Melbourne, Australia

Correspondence to

Dr Andrew Horne, MRC Centre for Reproductive Health, Queen's Medical Research Institute, 47 Little France Crescent, Edinburgh EH16 4TJ, UK

Tel: +44 131 242 6609 Email: andrew.horne@ed.ac.uk

Key words

Ectopic pregnancy, methotrexate, gefitinib

Abstract

Introduction: ~~Ectopic~~ Tubal ectopic pregnancy (tEP) is the most common life-threatening condition in gynaecology. T~~Small~~ EPs ~~(~~with pre-treatment serum human chorionic gonadotrophin [hCG] levels <10~~5~~00IU/L~~)~~ respond well to outpatient medical treatment with intramuscular methotrexate (MTX). T ~~Larger~~ EPs with hCG >1000IU/L take a significant time to resolve with MTX and require multiple outpatient monitoring visits. Gefitinib is an orally ~~-~~active epidermal growth factor receptor (EGFR) antagonist. In preclinical studies, we found that EP implantation sites express high levels of EGFR and that gefitinib augments MTX-induced regression of pregnancy-like tissue. We performed a Phase I toxicity study administering oral gefitinib and intramuscular MTX to 12 women with ~~small stable~~ tEPs. The combination therapy did not cause significant toxicities and was well ~~-~~tolerated. We noted that combination therapy resolved the tEPs faster than MTX alone. We now describe the protocol of a larger single arm trial to estimate the efficacy and side effects of combination gefitinib and MTX to treat ~~all~~ stable ~~ectopic pregnancies~~tEPs~~, including those~~ with hCG 1,000-10,000IU/L ~~of larger size currently considered unsuitable for medical therapy.~~

Methods and analysis: We propose undertaking a single-arm multicentre open label trial (in Edinburgh and Melbourne) and recruit 28 women with t~~large stable ectopic pregnancies~~EPs (pretreatment serum hCG ~~>~~1,0~~5~~00-10,000-IU/L). We will give a single dose of intramuscular MTX (50 mg/m2) and oral gefitinib (250mg) daily for seven days. Our primary outcome is resolution of ectopic pregnancy to non-pregnant hCG levels <15IU/L ~~within 25 days~~without requirement for surgery ~~to non-pregnant hCG levels <5 IU/L~~. Our secondary

For peer review only

outcomes ~~is~~ are comparison of time to resolution against historical controls given MTX only, and safety and tolerability as determined by clinical/biochemical assessment. ~~Outcomes will be compared to historical controls given methotrexate only.~~

Ethics and dissemination: Ethical approval has been obtained from Scotland A Research Ethics Committee (MREC 11/AL/0350), Southern Health Human Research Ethics Committee B (HREC 11180B) and the Mercy Health Human Research Ethics Committee (R12/25). Data will be presented at international conferences and published in peer-reviewed journals.

Trial registration number: ACTRN12611001056987.

For peer review only

Article summary

Article focus

Protocol of a study to determine:

- Is combination therapy with methotrexate and gefitinib ~~more~~ effective at resolving ~~ectopic pregnancies~~tEPs ~~than methotrexate alone~~?
- Is combination therapy with methotrexate and gefitinib safe and well ~~-~~ tolerated?

Key messages

- ~~Small stable ectopic pregnancies~~tEPs with hCG levels <1,000IU/L respond well to treatment with intramuscular methotrexate.
- ~~Larger ectopic pregnancies~~tEPs with hCG levels >1,000IU/L require multiple hospital visits to resolve with methotrexate and often require surgery.
- Novel combination therapy of methotrexate and the oral EGFR antagonist, gefitinib, could reduce the number of hospital visits required to resolve tEPs with hCG levels >1,000IU/L~~larger ectopic pregnancies~~.

For peer review only

Strengths and limitations of this study

- This is a Phase II exploratory efficacy trial, and will be the "first in man" to examine the efficacy of gefitinib and methotrexate to treat tEPs with hCG levels >1,000IU/Llarger ectopic pregnancies.
- This is a 'single arm' trial comparing outcome with historical controls. The data will be used to inform a future large multicentre randomised controlled trial comparing combination therapy to surgery for treatmentconventional management of ectopic pregnanciestEPs.
- The combination therapy described also has potential use in other pregnancy disorders where medical regression of placental tissue could be useful e.g. molar disease, retained products of conception after miscarriage, and regression of placenta accrete post-partum.

For peer review only

Introduction

~~Ectopic~~ Tubal ectopic pregnancy (tEP) is the most common life-threatening condition in modern gynaecology in both the developed and developing world (Sivalingham et al. 2011; Wilkinson and Jurkovic, 2011). ~~Small~~ TEPs ~~(~~with pre-treatment serum human chorionic gonadotrophin [hCG] levels <1,0~~5~~00IU/L~~)~~ respond well to outpatient medical treatment with an intramuscular injection of methotrexate (MTX). Indeed, it has been ~~recently~~ suggested that these ~~small ectopic pregnanciet~~EPs could be managed safely, and equally efficiently~~,~~ by expectant management without medical intervention (RCOG, 2010; Mavrelos et al, 2013; van Mello et al, 2013). In contrast, single-dose MTX is only cost–effective in women with serum hCG concentrations <1,500 IU/l (Mol et al. 2008)~~.~~, ~~for larger~~ In tEPs with higher hCG levels ~~(~~ ~~~~~(>60% of total tEPs), emergency laparoscopic surgical excision (with its inherent risks of damage to visceral organs) remains the most effective treatment. ~~(Mol et al. 2008).~~ T~~Larger~~ EPs with higher hCG levels take a significant time to resolve with MTX and require multiple outpatient monitoring visits. There therefore exists a need for more effective medical treatments for ~~larger~~ tEP~~s~~s with higher hCG levels to reduce the need for emergency surgery and reduce the time to resolution associated with MTX management.

Formatted: Not Highlight

Formatted: Not Highlight

Formatted: Not Highlight

Formatted: Highlight

Gefitinib is an orally active epidermal growth factor receptor (EGFR) antagonist licensed to treat non-small cell lung cancer (Herbst et al. 2004). In preclinical studies, we found that EP implantation sites express high levels of EGFR and that gefitinib augments MTX-induced regression of pregnancy-like tissue (Nilsson et

For peer review only

al. 2010). To translate this into clinical care, we performed a Phase I single-arm open-label dose-escalation study administering a combination of 250mg oral gefitinib (one dose [n=3], three daily doses [n=3], seven daily doses [n=6]) and intramuscular MTX (50 mg/m2) to 12 women with tEPs. The combination therapy did not cause any significant toxicities, and was well tolerated. We noted that resolution (fall in serum hCG to <15IU/L) with combination therapy was faster than the median time for tEPs to resolve with MTX alone when compared to contemporaneous controls (21 versus 32 days).

Objectives

The objective of this trial is to evaluate the efficacy and side effects of combination gefitinib and MTX to treat ~~larger~~ tEPs~~ectopic pregnancies~~ (~~pretreatment~~ hCG ~~>~~1,000-10,000IU/L~~1500~~)~~, including those currently considered unsuitable for medical therapy~~.

For peer review only

Methods and analysis

Study design

Phase II single-arm multi-centre open label trial (Edinburgh and two sites in Melbourne).

Subjects

~~40~~ 28 women with ~~large stable ectopic pregnancies~~tEPs with hCG levels 1,000-10,000IU/L.

Study settings

We will recruit patients from gynaecology departments within NHS Lothian (UK), and Southern Health and Mercy Health networks in Melbourne, Australia.

Sample size

We have calculated the sample size using A'Hern's formula for Phase II one-stage designs ~~[~~(A'Hern ~~RP (200~~1)~~1). Sample size tables for exact single stage Phase II designs. Statistics in Medicine, 20, 859-866]~~. For treatment of tEPs with hCG levels 1,000-10,000IU/L by methotrexate/gefitinib to be considered effective, we would expect a success rate of at least 90%. However, a success rate of 70% or less would be considered unacceptable. With 80% power and a 5% level of significance, 28 patients are required to enable us to assess whether the proportion of patients with a successful outcome to treatment is ≤ 70% or ≥90%. If 24, or more, patients have a successful outcome, we can reject the

For peer review only

hypothesis that the true efficacy of methotrexate/gefitinib is ≤ 70% and progress to a phase II trial.

~~required to estimate the true treatment effect with 90% confidence that the lower limit is at least 90%. We have assumed for the sake of these calculations that the true treatment efficacy of methotrexate/gefitinib in treating these tEPs with hCG levels 1,000-10,000IU/L larger ectopic pregnancies is 9855%, based on previously published data (Menon et al, 2007). We have chosen in this scenario, a sample size of 36 to ensure the lower limit of the 90% CI will exceed 90% (assuming a treatment effect of 9585%). Our sample size of 40 will thus ensure we have sufficient precision in estimation of the true treatment effect, allowing 10% loss to follow up. We believe that this is a reasonable basis on which to conduct this initial efficacy study, of methotrexate/gefitinib in the treatment of tEPs with hCG levels 1,000-10,000IU/Llarger ectopic pregnancies that are currently treated with surgery~~.

Formatted: [Normal], Line spacing: single, Tab stops: 2.01", Left + 4.03", Left + 5.54", Left

Formatted: Highlight

Formatted: Highlight

Formatted: Highlight

Formatted: Highlight

Formatted: Highlight

Formatted: Highlight

Formatted: Highlight

Inclusion criteria

Women aged ~~aged~~ between 18-45 years; pre-treatment serum hCG of ~~>~~1,000-10,000IU/L ~~1500 IU/L~~(rising or static); ultrasound diagnosis of definite tEP (extrauterine gestational sac with yolk sac and/or embryo, with or without cardiac activity) or probable tEP (inhomogeneous adnexal mass or extrauterine sac-like structure) (Barnhart et al, 2011) performed by a clinical team of trained, qualified and experienced ultrasonographers~~an abnormal mass outside the uterine cavity~~; no clinical evidence of intra-abdominal bleeding; no pallor; no guarding/rigidity on abdominal examination; stable blood pressure and heart rate; ~~normal~~ haemoglobin on full blood examination at day 1 between 100-165 g/L) .

Exclusion criteria

Women with a pregnancy of unknown location; evidence of a significant intra-abdominal bleed on ultrasound defined by free fluid above the uterine fundus or

For peer review only

the surrounding ovary (Fauconnier et al, 2007); wWomen with a history of any significant pulmonary disease; abnormal liver/renal/haematological indices; significant pre-existing dermatological conditions; significant pre-existing gastrointestinal medical illnesses; Japanese ethnicity.

Participant enrolment

All gynaecology consultants within NHS Lothian (UK), Southern Health and Mercy Health (both Australia) will be sent a letter informing them of the study and requesting permission to approach their patients. The clinical research team in NHS Lothian, Southern Health and Mercy Health will approach eligible women, provide them with patient information sheets and offer them the opportunity to discuss the trial, and obtain informed consent. Consent will only be taken once the patient has had ample time to read the patient information sheet and had her questions answered.

For peer review only

Intervention

Formatted: Line spacing: single

Eligible women will be given a single dose intramuscular methotrexate (50 mg/m2) injection with seven daily doses oral gefitinib (250 mg). The gefitinib will be started on the same date as the methotrexate injection is given.

Data collection

Data storage

A log with the patients' name and date of birth will be kept along with their unique study number in a separate file. All the data generated from the study will be stored in an anonymised form in a bespoke database, which will also be password protected. Only anonymised information will be stored on this, and participants will only be identifiable by their study number. All paperwork will be kept in a locked filing cabinet in a locked office. All data will be stored on university server (University of Edinburgh) on a password-protected computer with limited access to the research team, in accordance with the Data Protection Act (UK).

Screening

A member of the research team will carry out a screening visit to assess eligibility. All data will be recorded on a case record form and transferred to a secure database.

Participant log

For peer review only

The clinical research team will keep an electronic log of women who fulfil the eligibility criteria, women who are invited to participate in the study, women recruited and women who leave the trial early. Reasons for non-recruitment (eg, non-eligibility, refusal to participate, administrative error) will also be recorded. We will attempt to collect reasons for non-participation from women who decline to take part after previously providing contact details. During the course of the study, we will document reasons for withdrawal from the study and loss to follow-up.

Assessments

To monitor treatment response, we will follow protocols used clinically for single dosemedical management with methotrexate. Serum hCG levels will be measured on days 4, 7 and 11, then weekly until hCG levels drop to non-pregnant levels (<15 IU/L). Medical management will be discontinued and patients will undergo surgery based on their response to methotrexate and clinical picture (e.g. clinical evidence of intra-abdominal bleeding) following standard clinical paradigms documented by the assessing clinician. Participants will be contacted at 3 and 6 months post treatment to document return of menstrual cycles and any subsequent pregnancies. To monitor safety and tolerability, women will be assessed clinically (history) and biochemically (haematological, renal and liver function tests) on days 4, and 7 and 11, then weekly until the ectopic pregnancy has resolved (see below)(or if elevated, until return to normal physiological levels).

Primary outcome

For peer review only

Our primary outcome is resolution of ~~ectopic pregnancy~~tEP ~~within 25 days~~without requirement for surgery. Resolution is defined by serum hCG levels (the current clinical marker to monitor treatment response) falling to non-pregnant levels (hCG <15IU/L) ~~(<5 IU/L)~~. We have selected our primary outcome based on the data from our Phase I trial ~~that suggested that duration of resolution with combination therapy was faster than the median time for EPs to resolve with MTX alone (21 versus 32 days)~~where two patients recruited with pretreatment hCG levels >1,000IU/L required surgery and previously published data (Menon et al, 2007). ~~For practical purposes, we have chosen a primary outcome of resolution within 25 days rather than 21 days because patients are not normally seen between 18 and 25 days post treatment (our trial participants are followed up weekly from day 11 to conform to standard monitoring protocols for treatment with MTX) (RCOG 2004).~~ We are using a cut-off of <15IU/L, which corresponds to a negative urinary pregnancy test using the most sensitive assays.

Secondary outcome

(i) Time to resolution (categorical variable) compared to historical controls of similar pre-treatment serum hCG levels (identified by an individual blinded to the study).

(ii) Safety and tolerability as determined by clinical and biochemical assessment. Both methotrexate and gefitinib have the potential to affect haematological, renal and liver function.

Formatted: List Paragraph, Numbered + Level: 1 + Numbering Style: i, ii, iii, … + Start at: 1 + Alignment: Left + Aligned at: 0.25" + Indent at: 0.75"

Formatted: English (U.K.)

Proposed analyses

For peer review only - http://bmjopen.bmj.com/site/about/guidelines.xhtml

Given this is a single arm efficacy trial, the majority of the data will be expressed as descriptive statistics.

Ethics and dissemination

Ethical approval has been obtained from the Scotland A Research Ethics Committee (LREC 12/SS/0005) (UK), the Southern Health Human Research Ethics Committee B (SH HREC 11180B) and the Mercy Health Human Research Ethics Committee (R12/25) (both Australia). Data will be presented at international conferences and published in peer-reviewed journals. We will make the information obtained from the study available to the public through national bodies and charities (e.g. Ectopic Pregnancy Trust).

Adverse events

Participants will collect information about adverse events in their treatment diaries. However, they will be instructed to contact the clinical research team at any time after consenting to join the trial if they have an event that requires hospitalisation or an event that results in persistent or significant disability or incapacity. Any serious adverse events that occur after joining the trial will be reported in detail in the participant's medical notes, followed up until resolution of the event and reported to the ACCORD Research Governance (http://www.accord.ed.ac.uk) and QA Office based at the University of Edinburgh, or the Southern Health/Mercy Health Human Research Ethics Committees and Therapeutic Goods Administration of Australia's Office of Scientific Evaluation immediately or within 24-72 hours.

For peer review only

1

For peer review only

Discussion

If effective, we believe that this combination (gefitinib and methotrexate) could become standard of care for stable ~~ectopic pregnanciest~~EPs. The combination also has potential use in other pregnancy disorders. There may be other important conditions where medical regression of pregnancy tissue could be useful, for example, women with complete molar pregnancies and persistent molar disease, and ~~women with retained products of conception after miscarriage,~~ women with placenta accrete postpartum (to avoid hysterectomy)~~, and therapeutic termination of pregnancy~~.

Regarding the safety of gefitinib, data from post marketing surveillance representing over 92,000 patients exists, and has shown that EGFR inhibitors are well tolerated and largely free of serious side effects (FDA report: (Cohen et al, 2004) . Of note, the data on tolerability is based on patients taking gefitinib daily on an ongoing, indefinite basis, after primary treatment of a cancer. Diarrhea and skin rash are the most common side effects (20-30%). The skin rash, described as acneiform, can be severe, but is generally self-limited. Skin rashes occur within a month of initiation of treatment, but rarely in the first week. Interstitial lung disease (ILD) is a very rare but serious side effect of gefitinib. It is a thickening of the lung parenchyma that can be fatal in a third of cases. Of the 31,045 patients in the USA who took gefitinib (reported to the FDA), 84 developed ILD (0.3%). We plan to administer seven 250mg gefitinib tablets, one daily for only seven days, in addition to methotrexate. This is an extremely short duration of treatment compared with gefitinib's current marketing indications

For peer review only

and existing data usage. We would not expect this short course to have an adverse long-term effect on fertility but we will be assessing participants 3 and 6 months post treatment to document return of menstrual cycles and any subsequent pregnancies.

~~However, w~~We do not anticipate that this will be the final trial to determine whether further exploration of ~~this area~~combination therapy with gefitinib and methotrexate is worthwhile. We hope that the study will generate sufficient "signal" that gefitinib and methotrexate may be effective and safe, to support a funding application for a larger trial with a comparative group. Such a trial could be designed as an "equivalence" trial in terms of treatment efficacy between ~~surgery~~ conventional management and the gefitinib/methotrexate comparison. It would aim to test the hypothesis that gefitinib/methotrexate was superior in a range of outcomes prioritised by consumer groups and clinicians. We anticipate that these outcomes could include: ~~time in hospital,~~ time to resumption of normal activities, SF-36 at intervals after treatment, and patient satisfaction scores. Outcomes of a subsequent pregnancy are also important but would require long-term follow up studies. We anticipate that focus groups and surveys of patients and clinicians would be required to define the outcomes (other than efficacy) of these studies.

References

A'Hern RP. Sample size tables for exact single stage Phase II designs. Stat Med 2001 20:859-866.

For peer review only

Barnhart K, van Mello NM, Bourne T, Kirk E, Van Calster B, Bottomley C, Chung K, Condous G, Goldstein S, Hajenius PJ, Mol BW, Molinaro T, O'Flynn O'Brien KL, Husicka R, Sammel M, Timmerman D. Pregnancy of unknown location: a consensus statement of nomenclature, definitions, and outcome. Fertil Steril. 2011 95(3):857-66.

Cohen MH, Williams GA, Sridhara R, Chen G, McGuinn WD Jr, Morse D, Abraham S, Rahman A, Liang C, Lostritto R, Baird A, Pazdur R. United States Food and Drug Administration Drug Approval summary: Gefitinib (ZD1839; Iressa) tablets. Clin Cancer Res. 2004 10(4):1212-8.

Fauconnier A, Mabrouk A, Salomon LJ, Bernard JP, Ville Y. Ultrasound assessment of haemoperitoneum in ectopic pregnancy: derivation of a prediction model. World J Emerg Surg. 2007 2:23.

Herbst RS, Fukuoka M, Baselga J. Gefitinib-a novel targeted approach to treating cancer. Nat Rev Cancer 2004;4:956-65.

Jurkovic D, Wilkinson H. Diagnosis and management of ectopic pregnancy. BMJ 2011 342:d3397.

Mavrelos D, Nicks H, Jamil A, Hoo W, Jauniaux E, Jurkovic D. Efficacy and safety of a clinical protocol for expectant management of selected women diagnosed with

Formatted: Font: Not Bold
Formatted: Font: Not Bold
Formatted: English (U.S.)

For peer review only

a tubal ectopic pregnancy. Ultrasound Obstet Gynecol 2013 [epub ahead of print].

Menon S, Colins J, Barnhart KT. Establishing a human chorionic gonadotropin cutoff to guide methotrexate treatment of ectopic pregnancy: a systematic review. Fertil Steril. 2007 87(3):481-4.

Mol F, Mol BW, Ankum WM, van der Veen F, Hajenius PJ. Current evidence on surgery, systemic methotrexate and expectant management in the treatment of tubal ectopic pregnancy: a systematic review and meta-analysis. Hum Reprod Update 2008;14:309-19.

Nilsson U, Gano YE, Johns TG, et al. Epidermal Growth Factor Receptor Inhibition as a Novel Tablet-Based Treatment to Medically Cure Ectopic Pregnancy. Reprod Sci 2010; 17(Supplement):157A.

RCOG. The management of tubal pregnancy. Green top Guideline No 21, published in May 2004 (reviewed 2010). http://www.rcog.org.uk/files/rcog-corp/GTG21_230611.pdf

Sivalingam VN, Duncan WC, Kirk E, Shephard LA, Horne AW. Diagnosis and management of ectopic pregnancy. J Fam Plann Reprod Health Care. 2011 37(4):231-40.

For peer review only

van Mello NM, Mol F, Verhoeve HR, van Wely M, Adriaanse AH, Boss EA, Dijkman AB, Bayram N, Emanuel MH, Friederich J, van der Leeuw-Harmsen L, Lips JP, Van Kessel MA, Ankum WM, van der Veen F, Mol BW, Hajenius PJ. Methotrexate or expectant management in women with an ectopic pregnancy or pregnancy of unknown location and low serum hCG concentrations? A randomized comparison. Hum Reprod. 2013 28(1):60-7.

For peer review only

Funding

This work is supported by an NHMRC Grant (#1008276) to ST, TJ and EW, and an MRC Centenary Award (G0802808) to AH. The funders will have no role in the study design; collection, management, analysis and interpretation of data; writing of the report; and the decision to submit the report for publication.

Competing interests

AH is funded by an MRC Clinician Scientist Fellowship and MRC Centenary Award (G0802808) and holds grants from the Chief Scientist's Office Scotland (CZH/4/688) (HC co-investigator) and Wellbeing of Women. HC holds an MRC DCS Grant (G003611), is a co-applicant on an MRC Centre Grant (G1002033) and has research collaboration funding from Bayer Pharma AG. UN, TJ, and ST are joint holders of patents that relate to the use of EGFR inhibition in treating ectopic pregnancies. ~~AH and HC hold the University of Edinburgh Patent 'Identification of Ectopic Pregnancies' number 0712801.0.~~

Authors' contributions

AH, ST: research, contribution of original material, editing and approval of final manuscript; AD, MS: contribution of original material, editing and approval of final manuscript; MS, MR, CD, EW, HC, TJ, SB, JM, JN: editing and approval of final manuscript.

For peer review only

Figure legend

Figure 1. Flow of participants through the study.

For peer review only
